# Partial monosomy 18p and 21q due to a paternal reciprocal translocation leading to holoprosencephaly

**DOI:** 10.1038/s41439-025-00314-2

**Published:** 2025-05-30

**Authors:** Hiroko Wakabayashi, Ayumi Matsumoto, Sakiko Komori, Masahide Goto, Toshihiro Tajima, Aiko Sasaki, Takayoshi Matsumura, Takanori Yamagata

**Affiliations:** 1 Division of Clinical Trial, Clinical Investigation Center, Jichi Medical University Hospital, Tochigi, Japan; 2 Division of Cardiovascular and Genetics Research, Center for Molecular Medicine, Jichi Medical University, Tochigi, Japan; 3 Department of Pediatrics, Jichi Medical University, Tochigi, Japan; 4 Division of Obstetrics, Center for Maternal-Fetal, Neonatal and Reproductive Medicine, Center for Medical Genetics, National Center for Child Health and Development, Setagaya, Japan

**Keywords:** Paediatric neurological disorders, Neural tube defects

## Abstract

Here we report a patient with holoprosencephaly (HPE) associated with 45, XY,der(18)t(18;21)(p11.2;q21.3),-21 derived from a paternal balanced reciprocal translocation. Array comparative genomic hybridization analysis revealed 18p11.32-p11.21 and 21q11.2-q21.3 deletions. So far, nine cases of monosomy 18p with an unbalanced translocation (18;21) have been reported, four of which presented with HPE. Our case provides a detailed long-term clinical course and helps us to better understand these rare genetic events.

Holoprosencephaly (HPE) is estimated to occur in 1 in 16,000 live births, typically between the 18th and 28th day post-fertilization. It is a developmental disorder of the central nervous system formation and midline facial structures, caused by impaired left–right differentiation of the fetal forebrain^1^. HPE is classified into alobar, semi-lobar and lobar types, with the severity of facial malformations ranging from cyclopia to normal, correlating with the degree of central nervous system abnormalities. While patients with milder forms of HPE survive in adolescence or adulthood, severely affected patients do not survive beyond early infancy. Regarding the genetic background, 25–50% are due to chromosomal structural abnormality, such as trisomy 13, del(13q), dup(13q), del(18p), del(7)(q36), dup(3)(p24-pter), del(2)(p21) and del(21)(q22.3) and 18–25% are due to pathogenic variants of single genes, such as *SHH*, *ZIC2*, *SIX3*, *TGIF*, *PYCH1*, *CDON*, *GLI2*, *FOXH1* and *NODAL.* ^1,2^.

Chromosome 18p deletion is characterized by intellectual disability, growth retardation and craniofacial dysmorphisms. Approximately 10% of patients with this syndrome show symptoms of HPE^1,2^. About two-thirds of the cases are de novo, and one-third of cases are caused by a de novo unbalanced translocation^3^. The critical gene for HPE is *5*′*-TG-3*′*-interacting factor* (*TGIF*) gene located at 18p11.3 ^1,2^.

We report a patient presenting HPE with an unbalanced translocation of chromosomes 18 and 21, 45,XY,der(18)t(18;21)(p11.2;q21.3)dpat,-21 derived from a paternal balanced reciprocal translocation.

The proband was an 8-year-old boy. He was the second child of healthy nonconsanguineous parents, in their early 30s. His older sister was in good health. His parents had no spontaneous abortion. Cleft lip and palate defects were found at 23 weeks of gestational age, with no other abnormalities. He was born by vaginal delivery at 40 weeks gestation. His birth weight was 2,574 g (standard deviation (s.d.) −2.2.), his height was 46 cm (s.d. −2.1) and his occipitofrontal circumference was 31.8 cm (s.d. −1.4). The Apgar score was 8 at 5 min. Multiple malformations were found including cleft lip and palate, depressed nasal bridge, palpebral fissure upslanted, hypertelorism, widely spaced nipples, micropenis and overlapping fingers. The muscle tone was normal. On the 8th day after birth, he was diagnosed with lobar HPE by brain magnetic resonance imaging (Fig. 1a,b). He was also diagnosed with syndrome of inappropriate antidiuretic hormone and treated with antidiuretic hormone. At 5 months of age, he was diagnosed with West syndrome and treated with valproic acid and clonazepam. Inguinal hernia repair was performed at 1 year of age. At 2 years of age, Nissen fundoplication and gastrostomy were performed to address oral intake difficulties and gastroesophageal reflex. At 3 years of age, oxygen therapy was initiated for severe mixed sleep apnea, and a tracheostomy was performed at 8 years of age. Currently, he has spastic quadriplegia and is bedridden. He cannot control his head, but he moves his hands with some intention. He expresses his feelings by crying. He is being treated with valproic acid, topiramate, phenobarbital, clobazam and clonazepam. However, his seizures remain intractable, and he continues to experience partial seizures with eye deviation and secondary generalized seizures.

**Fig. 1:**
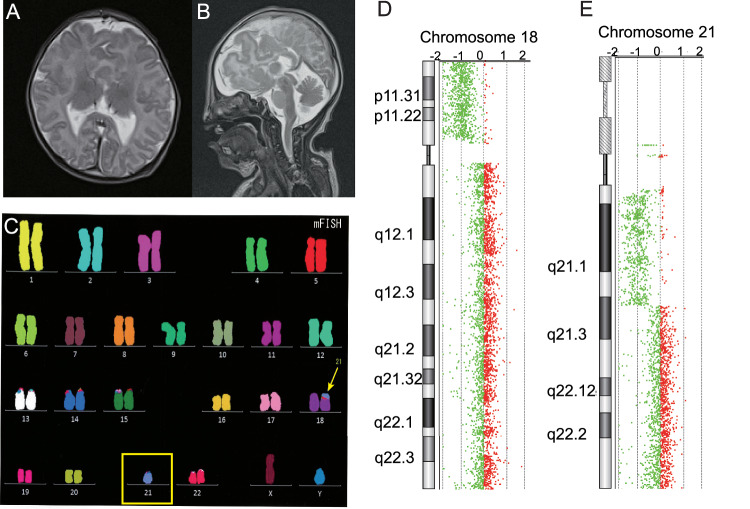
Clinical and genetic findings of the patient. **A, B** Brain magnetic resonance imaging at 8 days of age. T2-weighted axial **A** and sagittal **B** images show lobar HPE. **C** Multicolor FISH of the patient. An unbalanced translocation at 45,XY,der(18)t(18;21),-21 was revealed. The yellow box shows -21, and the arrow indicates der(18)t(18;21). **D, E** Array CGH of the patient. The vertical axis represents the chromosomal position, while the horizontal axis shows the fold change in copy number variation. Array CGH revealed 45,XY,der(18)t(18;21)(p11.2;q21.3),-21, 13.8-Mb deletion at arr[GRCh37] 18p11.32p11.21(131,700_13,935,908)×1 **D** and 12.7-Mb deletion at arr[GRCh37] 21q11.2q21.3(14,957,733_27,609,147)×1 **E**.

G-banding chromosome analysis showed 45,XY,-21. However, because full monosomy 21 is lethal, a chromosomal translocation was suspected. Multicolor fluorescence in situ hybridization (FISH) analysis revealed an unbalanced translocation at 45,XY,der(18)t(18;21),-21 (Fig. 1c). Chromosome 21q was found to be translocated to chromosome 18p, and a partial deletion of chromosome 18p was suspected. To elucidate the deletion of 18p and 21q, array comparative genomic hybridization (CGH) was performed using SurePrint G3 Human CGH Microarray Kit, 4×180K after written informed consent from his parents. The test was approved by the bioethics committee for human gene analysis at Jichi Medical University.

Array CGH revealed 45,XY,der(18)t(18;21)(p11.2;q21.3),-21 with 13.8-Mb deletion at 18p11.32-p11.21 (arr[GRCh37] 18p11.32p11.21(131,700_13,935,908)×1) and 12.7-Mb deletion at 21q11.2-q21.3 (arr[GRCh37] 21q11.2q21.3(14,957,733_27,609,147)×1) (Fig. 1d,e). The Online Mendelian Inheritance in Man (OMIM) map showed that 14 morbid genes, *THOC1*, *TYMS*, *SMCHD1*, *LPIN2*, *TGIF1*, *LAMA1*, *NDUFV2*, *APCDD1*, *PIEZO2*, *GNAL*, *TUBB6*, *AFG3L2*, *PSMG2* and *MC2R*, were deleted within the 18p11.32-p11.21, and that six morbid genes, *NRIP1*, *USP25*, *TMPRSS15*, *MRPL39*, *JAM2* and *APP*, were deleted within the 21q11.2-q21.3. Parental multicolor FISH analysis demonstrated that his mother’s karyotype was 46,XX, and that his father’s karyotype was 46,XY,t(18;21), indicating his father’s balanced reciprocal translocation.

Chromosome 18p deletion is known to cause intellectual disability, growth retardation, craniofacial dysmorphisms and HPE^1^. Chromosome 21q11.2-q21.3 deletion is associated with a range of clinical phenotypes, from normal development to various degrees of speech delay, global developmental delay and other developmental disorders^4–6^. In our patient, the deleted region was approximately 12.7 Mb in size, spanning from the central region to 27.6 Mb. A patient reported by Lindstrand et al. with a 14.0-Mb deletion from the central region to 28.2 Mb presented with borderline developmental delay, aortic stenosis and inguinal hernia^6^. Another case reported by Errichiello et al. involved a 14.5-Mb deletion from the central region to 27.5 Mb was associated with intellectual disability, global motor impairment, dysmorphic features such as a low hairline and widely spaced nipples, amenorrhea, obesity and pituitary microadenoma^5^. While the clinical features of our patient are largely attributable to the del(18p), the 21q11.2-q21.3 deletion may have a subtle effect on brain development and developmental delay. In addition, inguinal hernia and widely spaced nipples may also be explained by the 21q11.2-q21.3 deletion. Our patient did not present with hypotelorism or a single maxillary central incisor but instead exhibited cleft lip and palate defects along with hypertelorism (Table 1). Although hypotelorism is observed in approximately 80% of HPE cases^7^, complex facial clefts with hypertelorism, as seen in our patient, are also recognized features of HPE^8^.

**Table 1:** Nine previous cases and our case with monosomy 18p with an 18;21 translocation.

	Our case	Case 1	Case 2	Case 3	Case 4	Case 5	Cace 6	Case 7	Case 8	Case 9
Karyotype	45,XY,der(18) t(18;21)(p11.2;q21.3),-21	45,XY-18,-21,+der(18)	45,XY,-18,-21, +der(18)t(18;21) (p11.1;q11)	45,XY,-18,-21, +der(18)t(18;21)(p11.23;q11.2)	46,XX,-18,+ der(18)t(18;21) (p11.1;p11.1)	45,XY,dic(18;21) (q10;q10)	45,XY,dic(18;21)(q10;q10)/46,XY,del(18)(p11.1),del(21)(p11.1)	46,XY,der(18)t(18;21)(p11.2;q22.3)	45,XY,der(18)t(18;21)(p11.2;q22.1),-21	45,XX,-18,-21,t(18;21) (q11.1; q11.1)
Inheritance	Paternal	De novo	Maternal	De novo	Maternal	De novo	De novo	Maternal	De novo	De novo
Disjunction	3:1 segregation	3:1 segregation	3:1 segregation	3:1 segregation	Adjacent-1	3:1 segregation	3:1 segregation	Adjacent-1	3:1 segregation	3:1 segregation
HPE	+	−	−	−	−	+	+	+	−, polymicrogyria	+
Intellectual disability	+	−, IQ 80	+	−^a^	+	NA	NA	NA	NA	NA
Hypotelorism/hypertelorism	Hypertelorism	Hypertelorism	NA	−	NA	NA	Hypotelorism	Hypotelorism	−	−
Cleft lip and/or palate	+	−	NA	−	NA	NA	+	+	−	−
Others	Hypopituitarism			Hypopituitarism	Schizophrenia				Left hemi syndrome	Arrhinia, cyclopia
Follow up	8 years	10 years	8 months	3 years	43 years	Termination of pregnancy	17 days	Termination of pregnancy	11 months	Termination of pregnancy
Reference		Friedrich et al. (1982)^10^	Tharapel et al. (1991)^11^	Artman et al. (1992)^12^	Smith et al. (1996)^16^	Wang et al. (1997)^13^	Wang et al. (1997)^13^	Chen et al. (2001)^17^	Alkan et al. (2002)^14^	Goldstein et al. (2003)^15^

*NA* not available; *IQ* intellectual quotient.

^a^The Benet score was 99, but the patient showed specific learning disabilities in visual motor integration.

Reciprocal translocations lead to chromosomal imbalances by three types of disjunction: 3:1 segregation, adjacent 1 and adjacent 2 segregations. As chromosome 21 is acrocentric, 3:1 segregation is more likely to occur^9^. Previous reports of t(18;21) translocations and our case document eight instances of 3:1 tertiary monosomy^10–15^ and two instances of adjacent-1 segregation^16,17^ (Table 1). Among the eight cases with 3:1 tertiary monosomy, four presented with HPE. Their karyotypes are 45,XY,dic(18;21)(p11.1;p11.1)^13^, 45,XY,dic(18;21)(p11.1;p11.1)/ 46,XY,del(18)(p11.1),del(21)(p11.1)^13^, 45,XX,t(18;21)(q11.1;q11.1)^15^ and 45,XY,der(18)t(18;21)(p11.2;q21.3),-21 (our case). In addition, one case of adjacent-1 segregation, 46,XY,der(18)t(18;21)(p11.2;q22.3), was reported to have HPE^17^. The occurrence of HPE with 3:1 tertiary monosomy, four out of eight cases, is higher than the approximately 10% observed in overall 18p deletion syndrome. While a decrease in TGIF contributes to the development of HPE, other factors, such as disruptions in retinoic acid signaling, are also believed to be involved^18^. The precise mechanism underlying the higher frequency of HPE in 18p monosomy with 3:1 tertiary monosomy compared with overall 18p monosomy remains unclear and has yet to be elucidated. Among four previous cases of HPE, three cases were terminated^13,15,17^, and clinical course after infancy was not documented in the remaining case^13^. Our case highlights both the advancements and difficulties in managing HPE cases, and the long clinical course for 8 years described here will contribute to a better understanding of how to treat HPE cases in the future.

Concerning the inheritance, six instances were de novo^10,12–15^, three were maternally inherited^11,16,17^ and our case was paternally inherited (Table 1). As the father’s karyotype is expected to be 46,XY,t(18;21)(p11.2;q21.3) based on the proband’s karyotype, Stengel–Rutkowski’s method^19,20^ estimates the recurrence rate for this condition to be approximately 7% for paternal origin and 11% for maternal origin (Supplementary Table 1). However, precise estimation of the recurrent risk and genotype–phenotype correlation remains challenging due to limited data. Accumulating more cases is essential to establish a more accurate prediction method.

In summary, we present a boy with HPE and the karyotype 45,XY,der(18)t(18;21)(p11.2;q21.3),-21 caused by a paternal balanced reciprocal translocation. Our case helps us to better understand these rare genetic events.

## Supplementary information

Supplementary Table 1

## Data Availability

The relevant data from this Data Report are hosted at the Human Genome Variation Database at 10.6084/m9.figshare.hgv.350310.6084/m9.figshare.hgv.3506.
