# Supplementary material for: Partial monosomy 18p and 21q due to a paternal reciprocal translocation leading to holoprosencephaly
Source: Hum Genome Var. 2025 May 30;12:10. doi: 10.1038/s41439-025-00314-2 (PMC12125312; doi:10.1038/s41439-025-00314-2)
Supplement: Supplementary file 1 — Supplementary Table 1 [file 41439_2025_314_MOESM1_ESM.docx]

| Disjunction | Paternal | Maternal |
| --- | --- | --- |
| Adjacent-Ⅰ | 6.9 | 6.9 |
| Adjacent-Ⅱ | 0 | 0 |
| 3:1 disjunction type Ⅰ | 0 | 3.5 |
| 3:1 disjunction type Ⅱ | 0 | 0 |
| Interchange trisomy | 0.5 | 0.6 |
| Total risk (%) | 7.4 | 11 |

**Supplementary Table 1. The risk estimates for carriers of reciprocal translocations.** When the paternal karyotype is 46,XY,t(18;21)(p11.2;q21.3) (left) or the maternal karyotype is 46,XX,t(18;21)(p11.2;q21.3) (right), Stengel-Rutkowski's method estimates the recurrence rate to be approximately 7.4% or 11%, respectively. The interchanged trisomy risk is evaluated as follows. Trisomy 18: <0.2 (maternal) and <0.3 (paternal). Trisomy 21: 0.5 (maternal) and <0.6 (paternal). Therefore, the estimated risk is: Maternal risk: 0.1 (trisomy 18) + 0.5 (trisomy 21) = 0.6, and Paternal risk: 0.15 (trisomy 18) + 0.3 (trisomy 21) = 0.45^19^.
